# Safety of targeting tumor endothelial cell antigens

**DOI:** 10.1186/s12967-016-0842-8

**Published:** 2016-04-12

**Authors:** Samuel C. Wagner, Neil H. Riordan, Thomas E. Ichim, Julia Szymanski, Hong Ma, Jesus A. Perez, Javier Lopez, Juan J. Plata-Munoz, Francisco Silva, Amit N. Patel, Santosh Kesari

**Affiliations:** Batu Biologics, Inc, 9255 Towne Center Drive, #450, San Diego, CA 92121 USA; Medistem Panama, Panama City, Panama; Pan Am Cancer Treatment Center, Tijuana, Mexico; Medical School, Tecnologico de Monterrey, Monterrey, Mexico; Centre for Molecular and Cell-Based Therapeutics, SA de CV, Monterrey, Mexico; University of Utah School of Medicine, Salt Lake City, UT USA; John Wayne Cancer Centre, Santa Monica, CA USA

## Abstract

The mechanisms underlying discrimination between “self” and “non-self”, a central immunological principle, require careful consideration in immune oncology therapeutics where eliciting anti-cancer immunity must be weighed against the risk of autoimmunity due to the self origin of tumors. Whole cell vaccines are one promising immunotherapeutic avenue whereby a myriad of tumor antigens are introduced in an immunogenic context with the aim of eliciting tumor rejection. Despite the possibility collateral damage to healthy tissues, cancer immunotherapy can be designed such that off target autoimmunity remains limited in scope and severity or completely non-existent. Here we provide an immunological basis for reconciling the safety of cancer vaccines, focusing on tumor endothelial cell vaccines, by discussing the following topics: (a) Antigenic differences between neoplastic and healthy tissues that can be leveraged in cancer vaccine design; (b) The layers of tolerance that control T cell responses directed against antigens expressed in healthy tissues and tumors; and, (c) The hierarchy of antigenic epitope selection and display in response to whole cell vaccines, and how antigen processing and presentation can afford a degree of selectivity against tumors. We conclude with an example of early clinical data utilizing ValloVax™, an immunogenic placental endothelial cell vaccine that is being advanced to target the tumor endothelium of diverse cancers, and we report on the safety and efficacy of ValloVax™ for inducing immunity against tumor endothelial antigens.

## Leveraging the antigenic identity of tumors

The clinical possibility of manipulating the immune system to eradicate cancer originated in early work of William Coley who demonstrated tumor regression in soft tissue sarcoma patients treated with bacterial extracts in the early 1900s [1]. Based on observations that immune stimulation can be associated with spontaneous regression of cancer, numerous antigen-non-specific immunotherapy approaches have been introduced that are directed against cell-surface molecules, receptors, and immune effector pathways including Bacillus Calmette–Guérin (BCG), interleukin-2 (IL-2), interferons, CpG oligonucleotides, enzyme inhibitors targeting immune regulatory pathways, and antibodies against receptors involved in immune tolerance such as anti-CTLA-4 and anti-PD-1/PD-1 ligand antibodies [2]. Notably, these tactics for imparting generalized immune stimulation are capable of benefiting both innate and/or cell mediated components of anti-tumor immunity.

As knowledge concerning the exquisite specificity of the immune system has advanced, antigen-specific therapies are also being advanced to afford a degree of selectivity toward cancer cells. A groundbreaking area of immunotherapy has been the identification of antigens expressed by tumor cells and the epitopes thereof that elicit anti-tumor CD4+ and CD8+ responses. This line of investigation has revolutionized the field of immunotherapy through numerous breakthroughs including the following: (a) FDA approval for therapy with pre-primed antigen presenting cells (Provenge^®^) using a prostate cancer patient’s own dendritic cells to present a tumor antigen; (b) Immune checkpoint inhibitors designed to turn off inhibitory signals in the immune system and unmask effector T cell responses (for example, ipilimumab, nivolumab, pembrolizumab); and, (c) Promising results with genetically engineered chimeric antigen receptor (CAR)-T cells for addressing solid tumors. As will be described in more detail, the success of these strategies in terms of their immunogenicity against tumors was tempered with an element of caution owing to the complex relationship that exists between tumor immunity and autoimmunity.

The antigenic composition of tumors as well as tumor stromal and endothelial elements, and how this information can be utilized for vaccine design continues to be examined. These tumor-expressed antigens can be classed into several major categories that are not mutually exclusive [3]: (1) Over-expressed self-antigens found in both normal and neoplastic tissue; (2) Mutated tumor-specific antigens due to genetic mutations or alterations in transcription, or post-translationally modified antigens expressing; (3) Oncoviral antigens encoded by tumorigenic transforming viruses; (4) Oncofetal antigens that are normally only expressed during development and not in healthy adult tissues; (5) Lineage-specific antigens expressed by a particular tumor histotype; (6) Cancer testis antigens that are normally expressed by male germ cells and placental trophoblast; and, (7) Idiotypic antigens where the tumor expresses a specific clonotype, as occurs in leukemia and lymphoma. Significantly, tumor-specific antigens such as oncoviral antigens and mutated self-antigens have only been identified in some types of cancers, and the best-characterized targets of immunotherapy are the over-expressed self-antigens that may expressed at some level by non-malignant cells [4]. Indeed, T cells and antibodies from cancer patients recognize primarily tumor antigens shared in common with other self-tissues.

While knowing the composition of the tumor proteome is informative but these molecular criteria do not define the importance of individual tumor antigens as targets of therapy. Few tumor antigens are causal to the disease process and many are also not indispensable for cancer growth due to the array of redundant pathways that can be evoked for tumor growth, angiogenesis, and survival. Therefore, antigens that are useful as immunotherapeutic candidates must meet the criteria of encoding epitopes that are processed and presented to tumor-reactive CD4+ and CD8+ T cells and, furthermore, must invoke clinically beneficial responses (i.e. breaking tumor tolerance) without deleterious autoimmunity against healthy tissues [5].

Hand in hand with their varying degree of “self” identity, there are also theoretical differences in the ease with which T cell tolerance to these different categories of antigens can be overcome. On one end of the spectrum are antigens that are tumor-specific but are not found in normal tissue, for example, mutated oncogenes that could provide novel epitopes for T cell activation [5]. Owing to the fact that these antigens are not expected to trigger peripheral tolerance, the immunogenicity of these antigens is high and the risk of autoimmunity is low. Unfortunately, these antigens are not readily identifiable and are generally patient- or tumor type-specific. On the other hand, tumors contain a preponderance of antigens shared between tumor and normal tissues, which may evoke partial tolerance and activate low to intermediate affinity T cells. In this case, immunogenicity is lower; however, the theoretical risk for autoimmunity is greater [5]. Notably, peripheral tolerance against self-antigens is an imperfect process. High avidity T cells that respond to physiological quantities of a particular antigen are effectively deleted [5]. However, “self-ignorant” T cells that do not see their specific self-antigen in sufficient quantities or lower affinity T cells under normal conditions are often not eliminated and can persist in the periphery. The immunological players are thus in place for successful tumor vaccination; specifically, abundant self-antigen expression in tumors, and autoreactive T cells directed against these putative tumor rejection antigens.

## Degrees of “self” identity and tolerance thresholds to tumor antigen

Since tumors are essentially self-entities that possess the gamut of antigens from their tissue of origin, there are numerous layers of tolerance that immunotherapy must overcome. Soluble immunosuppressive mediators, including transforming growth factor beta and indolamine 2,3-dioxygenase (IDO), immune regulatory cells, receptor/ligand expression on tumor cells such as CTLA-4 and PD-1/PD-1L and tumor-derived exosomes all serve to dampen T cell activity (reviewed in [6]). In addition to the tumors themselves, the tumor vasculature is a promising immunotherapeutic target where factors such as hypoxia and over-expression of growth factor results in dysfunctional endothelial cells that are essential for tumor growth and survival [7]. Tumor endothelial cells express the death mediator Fas ligand, leading to loss of effector CD8+ T cells and a preponderance of tolerogenic regulatory T cells infiltrating into tumors [8]. Moreover, the tumor milieu contains an excess of vascular endothelial growth factor (specifically, VEGF-C, a lympho-angiogenic factor) that promotes apoptosis of tumor-specific CD8+ T cells in an animal model [9]. In a study of human cancer cell lines, VEGF was shown to prevent the maturation of dendritic cells, which is necessary for anti-tumor T cell activation [10]. If these barriers to T cell immunity can be overcome, anti-angiogenesis therapy is a powerful immunotherapy approach whereby it is conceptually possible for hundreds of tumor cells to be starved by knocking down a single tumor endothelial cell [11].

A model has been put forth that, for a given antigen, there is a critical threshold of immunity that must be exceeded for anti-tumor immunity or autoimmunity to be elicited [12]. In cancer, endogenous immunity against a particular tumor antigen is normally insufficient to overcome tumor-mediated immune suppression. The degree of stringency of tolerance for a specific antigen dictates whether a treatment strategy will raise immunity above this threshold. Thus, in response to vaccines incorporating numerous antigens such as whole cell vaccines, the critical thresholds of tolerance for each component antigen may differ. The key to effective vaccination is in activating the low-avidity, self-reactive T cells that have not been physically eliminated by peripheral tolerance to a level that can compensate for their relative weakness in recognizing tumor antigen [5]. Other antigen-independent factors including the choice of adjuvant, vaccine delivery method, and location of the tumor will also affect the outcome of attempts to boost T cell activity to an immunogenic level.

Past immunotherapeutic approaches have provided examples where breaking peripheral tolerance against over-expressed self-antigens present in both normal and neoplastic tissue antigen led to off-target toxicity. A well-studied example comes from paraneoplastic syndrome, a neurologic effect of cancer marked by loss of cerebellar Purkinje cells and pancerebellar symptoms that often manifest themselves before the cancer is identified [13]. Some patients with gynecologic cancer present with anti-Purkinje cell antibodies that are directed against tumor tissue as well as against the same antigens expressed in the cerebellum [14–16]. These antibodies have been proven to mediate cytotoxicity against cerebellar antigens [17]. Additionally, the neural antigen recoverin has been identified as an autoantigen in cancer-associated retinopathy (CAR), a paraneoplastic syndrome in which photoreceptors are targeted, leading to vision loss in some cancer patients [18].

Melanoma is one type of cancer in which the specificity of endogenous anti-tumor responses has been characterized in patients and cross-reactivity with healthy tissues has been documented. In the absence of immunotherapy, melanoma patients can spontaneously develop potent CD8+ T cell responses to melanocyte differentiation antigens. These T cell responses are associated with vitiligo, a cutaneous autoimmune disorder characterized by depigmentation of the skin, indicating the presence of potent anti-tumor immunity directed against antigens shared by normal melanocytes and melanoma cells [19]. Interestingly, the development of vitiligo in patients with melanoma is associated with an improved prognosis [20, 21]. Studies have identified that the melanoma-specific CD8+ T cells are reactive against tyrosinase, a melanosomal enzyme expressed during melanin biosynthesis in the skin [22]. Other targets of the immune response against melanoma were subsequently identified including TRP-1, TRP-2, and gp100, which are involved in melanin biosynthesis, and antigens like MART1/melan A that lack known function but are melanocyte-specific tissue differentiation antigens [23–26]. In fact, studies have shown that CD8+ T cell reactivity against melanocyte-specific differentiation antigens was the dominant response, whereas CD8+ reactivity against tumor-specific mutations was infrequent [27–30]. Hence, T cell reactivity against self-antigens can occur during naturally arising anti-tumor immunity in the absence of immunotherapy.

In melanoma, autoimmune sequelae resulting from immunotherapy appear to be increased in frequency and severity compared to those arising from natural immunity against tumors. Vitiligo has been frequently reported in response to immunotherapy including IL-2 therapy, adoptive transfers of tumor-infiltrating lymphocytes, and other tumor vaccines [6, 31]. A condition mimicking uveitis, an autoimmune reaction in the retinal pigment epithelium and the choroid of the eye, was reported in some melanoma patients undergoing immunotherapy with tumor-infiltrating lymphocytes plus IL-2, anti-CTLA-4 antibodies plus IL-2 with or without a gp100 vaccine [32–34]. Generally speaking, the more aggressive the immunotherapy regimen, the greater is the chance of autoimmunity resulting from activation of T cells specific for melanocyte differentiation antigens. It has been suggested that the incidence of autoimmune events, which included colitis, pruritus, dermatitis, hepatitis, hypophysitis and uveitis, were positively correlated with the dose of anti-CTLA-4 antibody administered [35]. To get a sense of the frequency of autoimmunity, a published study of 35 patients with metastatic melanoma undergoing a very aggressive treatment regimen consisting of lympho-depleting chemotherapy and then tumor infiltrating lymphocytes plus high-dose IL-2, the results revealed that twelve patients developed vitiligo, three patients had uveitis, and two patients were diagnosed as having both conditions. Although vitiligo and uveitis appear to be common effects of melanoma immunotherapy, these conditions are medically manageable, the latter complication being treatable by local steroid administration. Importantly however, other serious autoimmune manifestations resulting from melanoma immunotherapy have also been reported. In one study of 198 melanoma patients undergoing treatment with anti-CTLA-4 antibody, 21 % of the patients developed enterocolitis, requiring high dose systemic corticosteroid treatment, and five patients developed perforations or required colectomy [36]. Objective tumor response rates correlated with the autoimmune manifestations in the majority of patients. These examples demonstrate that autoreactive T cells do persist in the periphery and they can be awakened therapeutically as effectors with specificity for epitopes expressed by both tumors and healthy cells.

## Immunological rationale for vaccination strategies targeting the tumor vasculature

Vaccination strategies directed against tumor-associated endothelium are designed to take advantage of both quantitative and qualitative differences between tumor endothelial cells and non-malignant endothelial cells. An ideal tumor endothelial antigen is overexpressed in tumors and poorly expressed or absent in peripheral tissues. Given the high metabolic demands of tumors and their requirement for continued vascular development, tumor endothelial cells over-express an abundance of angiogenesis- and proliferation-associated molecules, in particular, numerous growth factors and their receptors. Putative vaccine targets that have been identified include tumor endothelial marker-1 (TEM1), vascular endothelial growth factor receptors 1 and 2, endoglin/CD105, basic fibroblast growth factor (bFGF) and its receptors, angiopoietin, and epidermal growth factor receptor (EGFR). While these antigens are over-expressed in tumor endothelium, they are also expressed to varying extents during physiological angiogenesis while being largely absent in quiescent endothelium of healthy adult tissues [37, 38]. In fact, systematic profiling of gene expression in tumor endothelium vs. healthy endothelium identified that the majority of tumor endothelium-associated genes could be detected in the angiogenic vessels of the ovaries, an indicator of physiological angiogenesis [38]. One notable exception is tumor endothelial marker 8 (TEM8), an integrin-like receptor that exhibits expressed restricted to tumors as well as human umbilical vein endothelial cells (HUVEC) where it is expressed during endothelial tube formation [38, 39]. Antigen profiling studies have also identified markers that are expressed on endothelium of select tumor types but not others [37]. Overall, the thrust of these studies has been to identify antigens found in abundance in tumor endothelium of diverse tumor types, thereby allowing for broader vaccine application, which also exhibit the lowest abundance or most restricted tissue distribution in non-malignant tissues.

Two general categories of tumor endothelial cell-based vaccines have been evaluated; namely, vaccines against specific peptides/proteins and/or whole cell vaccines consisting of endothelial cell lysates. Ultimately, the choice of antigen is one of numerous variables that must be optimized, given that the effectiveness for eliciting humoral and cell-mediated immunity against tumors is also affected by the mode of vaccine delivery (i.e. DNA, protein or peptide vaccines), the type of adjuvant, and the route(s) of vaccine administration [40]. A recent focus has been on clinical development of tumor endothelial cell-based vaccines consisting of whole cell lysates plus adjuvant stimulation for triggering anti-cancer immunity. By providing a myriad of antigens, polyvalent whole cell vaccines are believed to offer some advantages over monovalent (peptide) vaccines. Monovalent vaccines have potential drawbacks that include T cell epitope restriction to particular MHC haplotypes, inadequate activation of innate immunity, and the risk of immunoselection of epitope-loss variants [41]. On the other hand, the fact that polyvalent vaccines contain abundant intracellular antigens ubiquitous to all mammalian cells has raised the possibility that adverse autoimmune reactions might occur.

When considering a tumor endothelial cell-based vaccine from a safety perspective, a primary question is whether the polyvalent nature of a cell-based vaccine confers an autoimmunity risk, considering the fact that an array of structurally and functionally conserved endothelial cell antigens are present that are shared in common with healthy endothelium. As will be detailed below, the actual risk of autoimmunity has been low, as supported by numerous animal studies and preliminary clinical reports. The apparent safety of these vaccines may be attributable to the fact that only a finite antigenic component of a cellular vaccine is processed and presented to T cells—the epitope selection is restricted. Moreover, a theory has been put forth that self-reactive T cell responses arise against self-epitopes that are cryptic (i.e. previously hidden), a term that describes epitopes that are considered to be minor determinants, that are available in relatively low amounts after antigen processing [42]. Notably, T cells specific for cryptic epitopes are able to escape tolerance and persist in the periphery where they pose little harm to healthy tissues; however, they possess the potential to become activated under immunogenic conditions and when their cognate epitopes are available at higher concentrations [43]. In contrast, immunodominant epitopes are amply processed, allowing T cells that recognize them to be effectively deleted. If this theory is applicable, a cellular vaccine that provides sufficient antigen and immunogenic signals may activate tumor-reactive T cells directed against cryptic epitope with little risk of autoimmune manifestations elsewhere in the body.

In a study of a model self-antigen, it was demonstrated that exposure of antigen presenting cells to the cytokine IL-6 led to the processing and presentation of cryptic determinants of an antigen [44]. Display of cryptic determinants has been described as a cornerstone for activation of self reactive T cells in autoimmunity [45] and also been documented to occur in anti-tumor immunity [46]. Cryptic epitopes of self-antigens highly expressed by tumors have been demonstrated to be pivotal targets of therapy that result from qualitative and quantitative differences in antigen handling by APC [46]. IL-6 itself has a prominent role in tumor angiogenesis, upregulating VEGF expression [47]. Hyper-IL-6, an artificial fusion cytokine comprising IL-6 plus an artificial linker with the soluble IL-6 receptor, has been utilized as a component of whole cell vaccines against cancers [48]. On this basis, the choice of adjuvant use for delivery of a cancer vaccine can affect the epitopes that are processed and presented by antigen presenting cells, perhaps unveiling cryptic epitopes for T cell recognition. Indeed, one study suggested that both endogenous and exogenous influences, as dictated by the tumor microenvironment and the choice of vaccine, can overcome the default response of immunological ignorance to cancer [49].

## Safety of tumor endothelial cell-based vaccines

Table 1 outlines vaccines directed against tumor endothelium, specifically including protein/peptide vaccines that have been evaluated with respect to safety. The results demonstrate that, with the exception of one study, no effects were reported on wound healing and reproduction, two processes that involve endothelial cell proliferation and angiogenesis. To date, these approaches have been largely experimental and conducted in animals, although clinical trials have tested vaccination against vascular endothelial growth factor in cancer patients [50, 51]. Before any definitive conclusions can be drawn, safety of individual peptide vaccines against tumor vascular targets should be evaluated much more extensively in a clinical setting.

**Table 1:** Protein/peptide vaccines against tumor angiogenesis-associated antigens evaluated for safety in human and experimental studies

Vaccine	Tumor type	Adverse events/potential autoimmunity	References
Fibroblast growth factor (FGF)-2 peptide	Murine pulmonary metastatic cancer	No effect on wound healing, reproduction, organogenesis in offspring	[61]
Vascular endothelial growth factor receptor (VEGFR)-2 FLK1 DNA	Murine melanoma, colon carcinoma and lung carcinoma, metastatic pulmonary metastasis	No impairment of fertility, neuromuscular performance or hematopoiesis, slight delay in wound healing	[62]
VEGFR2 polypeptides, *Listeria monocytogenes* vector, microbial adjuvant, listeriolysin-O	Murine HER2 + breast cancer	No effect on wound healing or fertility	[63]
VEGF and *Neisseria meningitidis* outer membrane	Numerous in rats, rabbits, primates	No effect on skin deep wound healing	[64]
Endoglin DNA, *Salmonella typhimurium* vector	Murine tumors	No effect on wound healing	[65]
Survivin DNA *Listeria monocytogenes* vector	Murine pulmonary metastases of non-small cell lung carcinoma	No effect on wound healing or fertility	[66]
Tumor endothelial marker (TEM)-1 DNA fused to domain of the C fragment of tetanus toxoid	Numerous murine models	No effect on wound healing or reproduction	[67]
Notch ligand delta-like ligand 4 (DLL4) DNA	Murine mammary carcinoma	No effect on wound healing	[68]
CIGB-247, VEGF variant with bacterial adjuvant	Phase I clinical study of patients with advanced solid tumors	Only grade 1–2 adverse events	[50]
VEGFR2 peptide (VEGFR2-169) plus gemcitabine	Phase I clinical study of advanced pancreatic cancer	No severe adverse events, 83 % had immunological reaction at injection site	[51]
CIMAvax^®^ epidermal growth factor (EGF)	Human clinical study of patients undergoing surgery during treatment with CIMAvax^®^ EGF	No wound dehiscence, wound infection, delayed wound healing, fistula formation, abscess formation or bleeding associated with surgery during treatment with CIMAvax^®^ EGF occurred	[69]

Polyvalent vaccines comprised of tumor endothelial cell lysates that have been applied clinically have consisted of HUVEC and, more recently, a placental endothelial cell vaccines; ValloVax™. In the tumor milieu, the excessive release of angiogenic factors leads to over-expression of antigens that typify active and proliferating endothelium [38, 52–56]. Similarly, in both umbilical cord and placenta, in the face of the continually increasing demands for blood supply for the developing fetus, endothelial cells derived from these tissues are highly proliferative and angiogenic. In fact, fetal tissues are believed to immunologically resemble tumors, as first suggested by Dr. Valentin Govollo in the 1970s who noted the occurrence of immune cross-reactivity between placental trophoblast cells and lung cancer [57]. From an antigenic standpoint, the tumor endothelium is thought to more closely resemble endothelium from early fetal development than normal adult endothelium.

HUVEC have been used in pilot studies to test the anti-angiogenic effects as well as the safety of vaccination in patients with malignant brain tumors and metastatic colorectal cancer [58, 59]. In a published report where a total of 230 vaccinations were administered to a total of nine patients, MRI results showed partial or complete anti-tumor responses lasting for a minimum of 9 months in three of the patients with brain tumors [58]. Moreover, antibodies directed against HUVEC antigens were detected in eight out of total nine patients and HUVEC-specific CTL were detected in six of seven tested patients. No adverse events were reported with the exception of skin reactions at the vaccine injection site. In a related study where 352 vaccinations were performed in 17 patients with recurrent glioblastoma, reductions in tumor growth rates were observed and no adverse events were observed with the exception of skin reactions at the injection sites.

## Case study: treatment of cancer patients with ValloVax™, a novel tumor endothelial vaccine

ValloVax™ is a tumor endothelial cell vaccine consisting of placenta-derived endothelial cells pretreated, serving as a more abundant source of cells than umbilical cord, that are pre-treated with interferon gamma to enhance immunogenicity. In a previous pre-clinical study, we reported that ValloVax™ potently inhibited tumor growth in three histologically distinct animal models and also suppresses pulmonary metastasis subsequent to intravenous tumor administration [60]. In this report, we describe preliminary clinical safety and efficacy data, as measured on the basis of the ability of ValloVax™ immunization to elicit antibody response against well-established antigens that are over-expressed by tumor endothelial cells that are described in Table 2. Three patients with diverse types and stages of cancer that underwent treatment with ValloVax™ at the Pan American Cancer Treatment Center, under the Regenerative Medicine Institute Institutional Review Board approval, and follow up visits in the time period between May 2015 and October 2015.

**Table 2:** Tumor endothelial cell antigens investigated as targets of ValloVax™

Tumor endothelial target protein	Role of antigen in tumor angiogenesis	Refs.
Vascular endothelial growth factor receptor-1 (VEGFR-1); fms-like tyrosine kinase (flt)-1	Receptor that binds VEGF with high affinity Dominant receptor in the tumor microenvironment that is required for endothelial cell survival	[70, 71]
Vascular endothelial growth factor receptor-2 (VEGFR2); Flk-1, KDR	Also known as FLK-1 and KDR Binds to VEGF and is considered the most important receptor with regard to tumor angiogenesis Expressed on proliferating endothelium Low expression on healthy vascular endothelium	[62, 72]
Endoglin/CD105	Selectively expressed by endothelial cells undergoing vasculogenesis, angiogenesis, and inflammation Promotes endothelial cell proliferation, migration, and tube formation during early development and in tumors	[73–78]
Fibroblast growth factor receptor (FGFR)	Bind to fibroblast growth factor family members, having diverse roles in organogenesis, proliferation, pro-survival cell signaling and cellular migration FGF/FGFR system contributes to both adult angiogenesis and tumor angiogenesis FGFR signaling is implicated in escape of tumor vasculature from VEGF inhibitor treatment	[61, 79–82]
Integrin αvβ3; CD51/CD61	An extracellular matrix adhesion receptor that coordinates endothelial cell responses during angiogenesis Expressed in neo-angiogenic vessels; therefore, it is upregulated in the vasculature of solid tumors, while expression is low in quiescent vasculature	[83–87]
Tumor endothelial marker 1 (TEM1); endosialin or CD248	One of the most abundantly expressed proteins on tumor vasculature and tumor stroma of human tumors Expression is variable among tumor types; however, this protein can reportedly be expressed on endothelial cells, pericytes, and/or fibroblasts of human and animal tumors	[67, 88–93]

Patient 1 was a 55-year old male diagnosed with colon cancer and adjacent lymphadenopathy who had undergone colon resection and resection of extraperitoneal metastases. As per the standard protocol for ValloVax™ administration, patient 1 was treated with three subcutaneous injections of ValloVax™ on days 0, 7 and 14 and serum collection was performed pre-immunization and 12 and 22 weeks for detection of antibodies against tumor endothelial cell antigens in serum. The analysis for detection of antibodies against tumor endothelial antigens were conducted prior to immunization, and 4 and 10 weeks following immunization. Patient antibody analyses were conducted using ELISA techniques whereby plates were incubated in microwell plates with their respective capture proteins and dilutions of patient serum samples. The assay was performed using an OPD developing agent and optical density (OD) readings at 490 nm.

Patient 2, a 48-year old female, presented with rectal ulcerated moderately differentiated adenocarcinoma and a previous history of diffuse large non-B cell non-Hodgkins lymphoma. The patient was also treated with three subcutaneous injections of ValloVax™ on days 0, 7 and 14. Quantitation of antibodies against tumor endothelial antigens was performed using serum samples harvested at 10 and 16 weeks post-immunization to compare to pre-immunization serum.

Patient 3 was a 44-year old female with recurrent hemangioblastoma who previously underwent stereotactic radiosurgery and was administered ValloVax™ on days 0, 7 and 14. Serum samples taken pre-immunization were compared to those taken at one time point that was 7 weeks post-vaccination.

**Fig. 1:**
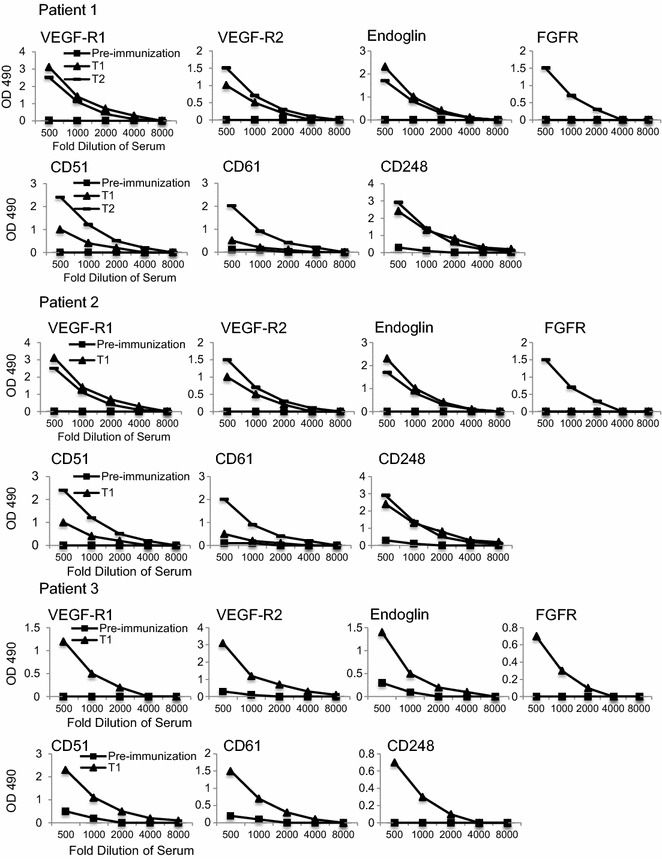
Analysis of antibodies against tumor endothelial antigens in three patients treated with ValloVax™. Antibodies against each of the antigens indicated were detected in dilutions of patient serum samples by ELISA and OD 490 readings were compared for pre-immunization, and time points (T1 and T2) following immunization with ValloVax™

The antibody analysis data for the three patients treated with ValloVax™ are presented in Fig. 1. Antibody responses against each of the tumor endothelial antigens examined, namely VEGF-R1, VEGF-R2, endoglin, FGFR, CD51, CD61 and CD248 (TEM-1, endosialin), were detected in virtually all the patients immunized with the ValloVax™ placental vaccine. In all cases, ValloVax™ immunization increased the concentrations of antibodies against these targets over pre-immunization levels. While these preliminary data do not attest to clinical outcomes of cancer patients, these experiments do reveal a specific immunoglobulin response against tumor endothelial antigens. As per Appendix, which comprises serum biochemistry panel results for these three patients for pre- and post-immunization time points, no abnormalities were noted. Additionally, no adverse events were reported associated with ValloVax™ administration based on medical examination and post-treatment interviews.

## Conclusions

Although tumors are comprised of self antigens that mirror the antigens expressed in normal tissues, and the elimination of autoreactive T cells is an incomplete process, the available evidence is in favor of the concept that anti-cancer immunity overlaps with but can be mechanistically uncoupled from deleterious autoimmunity. In designing novel immunotherapeutic strategies, many of the mechanisms for self-tolerance and epitope selection inherent to the immune system can operate in favor of selective anti-tumor immunity while minimizing the scope and severity of off-target toxicity. Novel cellular vaccines, including a placental endothelial vaccine, ValloVax™, have shown clinical promise in terms of safety and can hopefully be used to efficaciously guide the immune response toward destruction of pathological endothelial cells that are required for tumor growth and survival. Preliminary clinical data demonstrate that vaccination of cancer patients with ValloVax™ is not only well-tolerated but also elicits humoral immunity against an array of tumor endothelial cell-associated antigens with known pathological roles in tumor angiogenesis.

## Appendix

See Tables 3, 4 and 5.

**Table 3:** Patient 1

Test	Pre-immunization	Time point 1	Time point 2	Normal ranges
Bicarbonate (total) (mEq/L)	22	24	20	18–30
Calcium (total) (mg/dL)	10	10	11	9–11
Chloride (mEq/L)	99	101	103	98–106
Magnesium (mg/dL)	3.2	3.1	3.5	1.8–3.6
Phosphorus (mg/dL)	3.1	3.5	3.9	3–4.5
Sodium (mEq/L)	145	138	135	135–147
Alkaline Phosphatase (U/L)	140	79	74	50–160
Amylase (U/L)	122	119	110	53–123
Creatine kinase (U/L)	101	115	99	38–174 (males) 96–140 (females)
Lipase (U/L)	34	58	27	10–150
ALT (GPT) (U/L)	11	25	14	0–30
AST (GOP) (U/L)	39	24	12	0–40
Albumin (g/dL)	3.8	3.5	4.8	3.5–5.5
Bilirubin (total) (mg/dL)	.3	.4	.2	<1.0
Cholesterol (mg/dL)	202	183	222	<225
Creatinine (mg/dL)	1.7	1.5	1.1	1.0–2.0
Globulin (g/dL)	1.5	1.7	1.8	1.5–3.5
Glucose (mg/dL)	132	110	93	80–120
Protein (total) (g/dL)	5.8	6.5	6.1	6.3–8.0
Triglycerides (mg/dL)	226	221	232	40–200
Urea (mg/dL)	22	27	24	20–40
Uric acid (mg/dL)	2.5	2.2	2.1	2.0–4.0

**Table 4:** Patient 2

Test	Pre-immunization	Time point 1	Time point 2	Normal ranges
Bicarbonate (total) (mEq/L)	28	24	27	18–30
Calcium (total) (mg/dL)	11	10	10	9–11
Chloride (mEq/L)	105	101	102	98–106
Magnesium (mg/dL)	3.1	2.4	2.1	1.8–3.6
Phosphorus (mg/dL)	3.3	3.2	3.1	3–4.5
Sodium (mEq/L)	138	143	141	135–147
Alkaline Phosphatase (U/L)	154	144	164	50–160
Amylase (U/L)	104	121	120	53–123
Creatine kinase (U/L)	131	79	74	38–174 (males) 96–140 (females)
Lipase (U/L)	75	89	125	10–150
ALT (GPT) (U/L)	25	29	21	0–30
AST (GOP) (U/L)	13	36	36	0–40
Albumin (g/dL)	4.3	5.3	4.2	3.5–5.5
Bilirubin (total) (mg/dL)	.1	.2	.3	<1.0
Cholesterol (mg/dL)	95	201	199	<225
Creatinine (mg/dL)	1.6	1.7	1.5	1.0–2.0
Globulin (g/dL)	1.8	3.9	3.2	1.5–3.5
Glucose (mg/dL)	94	102	108	80–120
Protein (total) (g/dL)	7.3	8.4	7.5	6.3–8.0
Triglycerides (mg/dL)	185	174	186	40–200
Urea (mg/dL)	38	40	23	20–40
Uric acid (mg/dL)	3.1	3.9	3.4	2.0–4.0

**Table 5:** Patient 3

Test	Pre-immunization	Time point 1	Normal ranges
Bicarbonate (total) (mEq/L)	19	20	18–30
Calcium (total) (mg/dL)	12	11	9–11
Chloride (mEq/L)	105	104	98–106
Magnesium (mg/dL)	2.4	2.7	1.8–3.6
Phosphorus (mg/dL)	3.1	3.1	3–4.5
Sodium (mEq/L)	149	151	135–147
Alkaline Phosphatase (U/L)	111	159	50–160
Amylase (U/L)	108	80	53–123
Creatine kinase (U/L)	112	142	38–174 (males) 96–140 (females)
Lipase (U/L)	101	125	10–150
ALT (GPT) (U/L)	53	42	0–30
AST (GOP) (U/L)	84	42	0–40
Albumin (g/dL)	3.3	4.3	3.5–5.5
Bilirubin (total) (mg/dL)	.6	.7	<1.0
Cholesterol (mg/dL)	102	124	<225
Creatinine (mg/dL)	1.3	1.8	1.0–2.0
Globulin (g/dL)	2.4	3.2	1.5–3.5
Glucose (mg/dL)	114	101	80–120
Protein (total) (g/dL)	7.4	5.3	6.3–8.0
Triglycerides (mg/dL)	101	121	40–200
Urea (mg/dL)	29	32	20–40
Uric acid (mg/dL)	2.5	2.1	2.0–4.0
